# Optimization Function for Determining Optimal Dose Range for Beef and Seed Potato Irradiation

**DOI:** 10.3390/foods13233729

**Published:** 2024-11-21

**Authors:** Elena Kozlova, Ulyana Bliznyuk, Alexander Chernyaev, Polina Borshchegovskaya, Arcady Braun, Victoria Ipatova, Sergey Zolotov, Alexander Nikitchenko, Natalya Chulikova, Anna Malyuga, Yana Zubritskaya, Timofey Bolotnik, Anastasia Oprunenko, Aleksandr Kozlov, Mikhail Beklemishev, Roza Yagudina, Igor Rodin

**Affiliations:** 1 Department of Medical and Biological Physics, I.M. Sechenov First Moscow State Medical University (Sechenov University), 119991 Moscow, Russia; waterlake@mail.ru (E.K.); fillnoise@mail.ru (A.K.); 2 Department of Physics, Lomonosov Moscow State University, GSP-1, 1-2 Leninskiye Gory, 119991 Moscow, Russia; a.p.chernyaev@yandex.ru (A.C.); alexeevapo@mail.ru (P.B.); zolotov.sa15@physics.msu.ru (S.Z.); nikitchenko.ad15@physics.msu.ru (A.N.); yyryana@gmail.com (Y.Z.); 3 Skobeltsyn Institute of Nuclear Physics, Lomonosov Moscow State University, GSP-1, 1-2 Leninskiye Gory, 119991 Moscow, Russia; ipatova.vs15@physics.msu.ru; 4 Department of Chemistry, Lomonosov Moscow State University, GSP-1, 1-3 Leninskiye Gory, 119991 Moscow, Russia; avbraun@yandex.ru (A.B.); timab@tut.by (T.B.); oprunenko_anastasiya@mail.ru (A.O.); beklem2@inbox.ru (M.B.); igorrodin@yandex.ru (I.R.); 5 Siberian Federal Scientific Center of Agro-Biotechnologies, Russian Academy of Sciences, Novosibirsk Oblast, 630501 Krasnoobsk, Russia; natalya-chulikova@yandex.ru (N.C.); anna_malyuga@mail.ru (A.M.); 6 Department of Organization of Medical Provision and Pharmacoeconomics, I.M. Sechenov First Moscow State Medical University (Sechenov University), 119435 Moscow, Russia; yagudina_r_i@staff.sechenov.ru; 7 Department of Epidemiology and Evidence-Based Medicine, I.M. Sechenov First Moscow State Medical University (Sechenov University), 119991 Moscow, Russia

**Keywords:** food irradiation, tenderloin beef, naturally infected seed potato tubers, electron beam, X-rays, bacteria, myoglobin, albumin native structure, phytopathogen

## Abstract

The objective of this study is to develop a universally applicable approach for establishing the optimal dose range for the irradiation of plant and animal products. The approach involves the use of the optimization function for establishing the optimal irradiation dose range for each category of plant and animal product to maximize the suppression of targeted pathogens while preserving the surrounding molecules and biological structures. The proposed function implies that pathogens found in the product can be efficiently suppressed provided that irradiation is performed with the following criteria in mind: a high irradiation dose uniformity, a high probability of irradiation hitting pathogens and controlled heterogeneity of radiobiological sensitivity of pathogens. This study compares the optimal dose ranges for animal and plant products using beef tenderloin and seed potato tubers as examples. In a series of experiments, our team traced the dose dependencies of myoglobin oxidation in beef and the amount of potential damage to albumin’s native structure. The behavior patterns of myoglobin derivatives and the amount of potential damage to albumin found in this study determined the optimal dose range, which appeared to be wider for beef irradiation compared to that for seed potato tubers, as they do not require uniform irradiation of the entire volume since targeted phytopathogens are predominantly found within the surface layers of the tubers. The use of proprietary methods involving spectrophotometry and high-performance liquid chromatography–mass spectrometry provides a novel perspective on the quantitative assessment of the myoglobin oxidation level and the potential damage to albumin’s native structure.

## 1. Introduction

According to the Sustainable Development Goals set by the UN [1,2,3] to ensure global food safety and security, non-thermal methods of industrial food processing, such as food irradiation, are primarily used to combat a wide range of pathogens, which are increasingly becoming more resistant to different impacts in the changing natural environment [4,5,6]. Moreover, the food industry these days gives preference to non-thermal methods of food processing since such methods, especially irradiation, help to retain more nutrients compared with thermal methods that can degrade essential nutrients, without causing a negative impact on taste and texture of the food product. Considering the vast number of benefits of food irradiation, its popularity has been growing over recent years and now encompasses the irradiation of spices, root crops, fruits and vegetables on an industrial scale [7]. It should be noted that food irradiation is essential for ensuring the safety of retort products, processed and packaged in a way that obviates the need for the refrigeration of emergency food [8], space food [9], medical diets for immunocompromised patients [10] as well as providing food security and safety in the case of the long-haul transportation of foods.

While industrial food irradiation is regulated by international standards [11], they fail to address the needs of specific categories of product which require a fine dose adjustment due to their complex and diverse content. Meat, for example, contains a variety of fats, proteins and carbohydrates [5,12,13], which make this category of product highly susceptible to bacterial contamination, so a special approach is required for choosing the dose range that would prevent undesired changes in the composition of meat tissues. When it comes to dose adjustment, meat represents a certain challenge since it contains a wide variety of pathogens, namely, various strains of *Escherichia coli*, *Salmonella*, *Listeria monocytogenes*, *Shigella*, *Campylobacter*, *Brucella*, *Mycobacterium bovis*, as well as toxins produced by *Staphylococcus aureus*, *Clostridium* species and *Bacillus cereus* [14,15,16,17,18,19,20,21], which show different radiosensitivities depending on the type of product, temperature and pH [22]. For example, while the dose range 0.56–0.62 kGy reduces *Salmonella* content by a factor of 10 in pork when pork is irradiated with photons generated by radioisotope ^137^Cs, raw rice requires 0.234–0.266 kGy [23,24] to suppress *Salmonella* to the same level. Similarly, the dose range 0.232–0.236 kGy is sufficient for inhibiting *Listeria monocytogenes* in rice; however, smoked turkey requires a higher dose 0.58 kGy to suppress *Listeria monocytogenes* to the same level [25]. Anaerobic bacteria, such as *Clostridium Botulinum*, are significantly more resistant to irradiation, as they form spores that can only be suppressed with much higher doses, ranging from 1.3 kGy to 3 kGy, depending on the strain [26].

The type of pathogens is not the only factor which has an impact on the suppression efficiency of microorganisms. Another key factor is the type of irradiation and irradiation parameters, including the energy spectrum, dose uniformity and linear energy transfer values in the irradiated product. For instance, *Escherichia coli* in rice can be suppressed ten-times by 0.228–0.367 kGy when rice is irradiated with photons generated by radioisotope ^60^Co, whereas bremsstrahlung irradiation suppresses *Escherichia coli* in rice to the same level with a dose of 0.403 kGy [23]. All these factors add up to the complexity of determining optimal dose ranges for different categories of foods.

Optimal dose ranges, however, should represent a balance between two goals—the suppression of pathogens and the preservation of organoleptic properties of the foods, which depend on the condition and content of fats, proteins and carbohydrates in the irradiated product. Undesired changes in the taste of the product primarily occur as a result of chemical transformations of lipids, amino acids and carbohydrates initiated by free radicals and organic radicals occurring in the product due to water radiolysis [12,27,28,29]. Both types of radicals initiate lipid oxidation, which leads to the formation of a large number of different high- and low-molecular-weight compounds, particularly volatile organic compounds. Volatile organic compounds, which are predominantly oxidative products of lipids [3,27,30], are elusive and prone to chemical transformations as a result of irradiation, so the change in macromolecules in meat due to irradiation can be detected by the change in the structure of proteins. This change can be traced using high-performance liquid chromatography–mass spectrometry (HPLC-MS) as this method allows one to identify the amount of damage to proteins as a result of irradiation. Irradiation causes functional changes in proteins [31,32]. The functional changes in proteins can be traced using confocal Raman spectroscopy [33] and high-performance liquid chromatography—mass spectrometry (HPLC-MS) as these methods allow one to identify the change in the configuration of protein native structure as a result of the impact of different chemical and physical factors, such as irradiation [34]. Spectrophotometry can also be used to trace the irradiation-induced functional changes in myoglobin present in large quantities in muscle fibers, since transformations between its derivatives are highly sensitive to any physical and chemical impacts [13].

On the other hand, naturally infected seed material, such as seed potatoes, has its own specific requirements to irradiation since the objective of irradiation is to reduce the amount of phytopathogens without a detrimental effect on the potato yield [35,36,37]. Since phytopathogens can be predominantly found in the surface layers of potato tubers [38], the radiation targeting the surface layers in order to suppress phytopathogens inevitably damages potato sprouts and potato flesh responsible for potato yield. Considering such conflicting goals of irradiation, the approach to establishing the optimal dose range and the method for efficient irradiation of seed potatoes should be flexible enough to factor in the diverse properties of seed potato varieties, such as the depth at which sprouts are located, antioxidant content, starch and sugar content, as well as the type and contamination of phytopathogens that can be found in potato tubers.

What makes the search for optimal irradiation parameters for different categories of food products even more complicated is the fact that pathogen suppression efficiency and the extent of the damage to surrounding macromolecules depend on the irradiation dose [39,40] and linear energy transfer (LET) values [41,42]. Considering that the dose and LET distributions in food products depend on the type of irradiation and its energy spectrum [3,43,44,45] as well as the density and chemical composition of the product [3,46], the approach to establishing optimal irradiation parameters should take into account the irradiation dose uniformity and LET distribution throughout the food product.

This interdisciplinary research focuses on developing a universally applicable approach to establishing the optimal dose range for the irradiation of plant products and animal products that encompasses both the physical parameters of irradiation and the nature of interaction between the irradiation and the substance of the food product, as well as the individual properties of different categories of foods.

This study analyzes the pathogen suppression efficiency and the extent of the damage to the molecules and cells that determine the nutritional value of the products, which are further referred to as “the surrounding molecules” or “the surrounding structures”.

This study compares how bacteria and proteins in animal products and phytopathogens and cells in plant products respond to irradiation. Seed potato tubers and tenderloin beef were selected for this study as examples of a plant product and an animal product to illustrate how the proposed approach addresses diverse problems posed by food irradiation. Seed potato tubers naturally infected with *R. Solani* represent a good example of how the proposed optimization function can be used to determine the optimal irradiation dose range to inactivate phytopathogens typically found close to the surface of potato tubers while minimizing negative impacts to the potato sprouts and flesh responsible for the yield. In the case of tenderloin beef, however, the optimization function is applied to find the dose range, which would cause a suppression of bacteria in the whole volume of tenderloin beef samples, with a minimum impact on proteins accountable for the nutritional value of the product.

To investigate the influence of irradiation dose uniformity and LET distribution throughout the food product on the pathogen suppression efficiency and the extent of the damage to surrounding molecules and cells, we study the impact of accelerated electrons and X-ray irradiation having a variable energy spectrum on the survival rate of bacteria in meat, pure cultures of *R. Solani* as well as on myoglobin derivatives and the albumin native structure.

## 2. Materials and Methods

### 2.1. Beef Irradiation Methodology to Find Optimal Dose Range for Beef Irradiation

#### 2.1.1. Objects of This Study

Tenderloin beef purchased at a local market was subjected to a microbiological analysis to determine the efficiency of microorganism inactivation in the beef samples after irradiation with accelerated electrons and X-ray irradiation. In order to ensure a uniform microorganism suppression, tenderloin beef homogenate was prepared as a 1:2 solution in 0.9% saline. The concentration of viable cells in the homogenate after dilution with saline solution was (1.0 ± 0.2) × 10^4^ CFU/g. The homogenate in the volume of 0.5 mL was put into 2 mL Eppendorf cylindrical tubes to ensure a uniform one-side electron beam irradiation and two-side X-ray irradiation.

Since beef has a high myoglobin content, the spectrophotometry method was found to be suitable for estimating myoglobin derivative concentrations in the beef samples. The 20 mm × 20 mm × 6 mm parallelepiped beef samples were put into Ø 35 mm Petri dishes for two-side electron beam irradiation.

To determine structural changes in proteins after irradiation, the 0.5 mg/L suspension of bovine serum albumin (BSA fraction V, BioClot) in 0.9% saline solution was used to estimate the concentration of the peptides selected from albumin amino acid sequence after the trypsin hydrolysis procedure. The concentrations of the selected peptides were measured using high-performance liquid chromatography–mass spectrometry (HPLC-MS). The 0.5 mL suspension was put into 2 mL Eppendorf cylindrical tubes to make sure that the samples were irradiated uniformly from one side with accelerated electrons and X-ray irradiation.

To compare the effects of accelerated electrons and X-rays on target microorganisms and proteins for determining the optimal dose range for beef irradiation, 72 homogenate beef samples, 108 albumin suspensions and 48 beef pieces were prepared; 30 homogenate beef samples, 48 albumin suspensions and 40 beef pieces were irradiated with electron beam; 30 homogenate beef samples and 48 albumin suspensions were irradiated with X-ray irradiation, while other samples were used as controls.

#### 2.1.2. Research Stages

With an aim to develop an algorithm for determining the optimal dose range for products of animal origin, which, on the one hand, effectively suppresses microorganisms and, on the other hand, minimally affects the surrounding molecules, after treatment with accelerated electrons and X-rays, meat samples were subjected to microbiological analysis, and the degree of oxidation of protein molecules was assessed by examining myoglobin derivatives and damage to the native structure of albumin.

**Figure 1:**
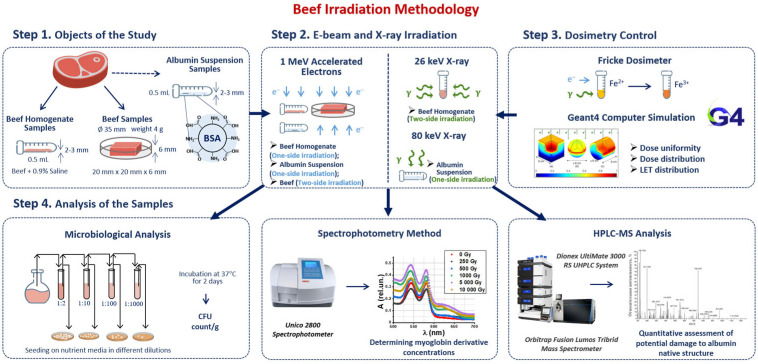
Research stages to determine the optimal dose range for beef irradiation.

Our methodology consisted of four steps involving sample preparation, irradiation, dosimetry control and a three-phase analysis of the irradiated and non-irradiated samples (Figure 1). Beef homogenate samples were irradiated with doses ranging from 250 Gy to 3000 Gy to compare the efficiency of 1 MeV electron beam (E-beam) and 26 keV X-ray irradiation for suppression of mesophilic aerobic and facultative–anaerobic microorganisms commonly found in beef (Figure 1 Step 2). To estimate the dose absorbed by the samples during irradiation, Fricke dosimetry was used for all samples. GEANT 4 computer simulation was carried out to determine the dose uniformity in the samples during E-beam and X-ray irradiation (Figure 1 Step 3). After irradiation, beef homogenate was subjected to the microbiological analysis by quantifying the number of viable cells in irradiated and non-irradiated homogenate. To study the transformations of myoglobin derivatives in beef during irradiation, beef pieces were irradiated with E-beam with the doses ranging from 250 Gy to 5000 Gy and then analyzed using spectrophotometry. BSA suspensions were irradiated with 1 MeV E-beam and 80 keV X-rays to compare the impact of electrons and bremsstrahlung photons on the extent of the damage to BSA native structure using trypsin hydrolysis and high-performance liquid chromatography–mass spectrometry (HPLC-MS) (Figure 1 Step 4).

#### 2.1.3. Electron Beam Irradiation

Beef homogenate, beef pieces and bovine serum albumin suspensions were irradiated with accelerated electrons having the energy spectrum represented in Figure 1, generated by 1 MeV electron accelerator UELR-1-25-T-001 (Skobeltsyn Institute of Nuclear Physics at Moscow State University, Russia) with an average beam power of 25 kW. During the irradiation, the beam current was 0.5 µA, and the ambient temperature was 20 °C.

**Figure 2:**
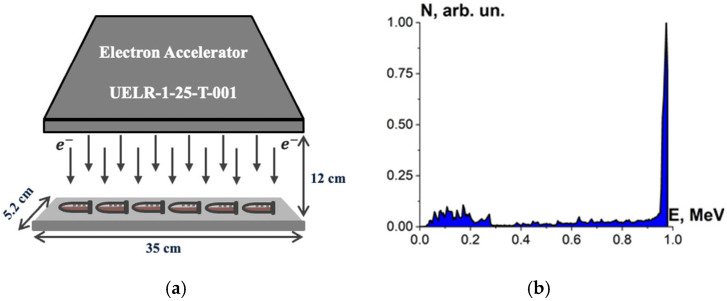
(**a**) Electron beam irradiation method using UELR-1-25-T-001 accelerator; (**b**) electron beam energy spectrum.

Six Eppendorf cylindrical tubes with beef homogenate, five cylindrical tubes with bovine serum albumin suspensions and eight beef pieces in Petri dishes were put on a 35 cm × 5.2 cm duralumin plate and irradiated for each irradiation session. During one-side irradiation, the samples were located in 12 cm far from the beam output (Figure 2a). The beef homogenate, bovine serum albumin suspensions and the beef pieces were irradiated in separate irradiation sessions: 5 sessions for the beef homogenate, 8 sessions for the bovine serum albumin suspensions and 5 sessions for the beef pieces. The beef homogenate and the bovine serum albumin suspensions were put on the duralumin plate 12 cm away from the beam output and subjected to one-side irradiation (Figure 2a). The spectrum of electron beam is shown in Figure 2b. The thickness of the homogenate and albumin suspension layer was (2.0 ± 0.5) mm. The beef pieces were irradiated from two opposite sides.

During irradiation, the charge *Q_exp_* absorbed by the part of the duralumin plate not occupied by the samples was registered to determine the dose absorbed by the samples using an analog-to-digital converter (OOO Industrial Association “Oven”, Russia), and the margin of error in determining the charge was no more than 2%.

#### 2.1.4. X-Ray Irradiation

To select the most effective radiation source for efficient inactivation of microorganisms, beef homogenate was irradiated with X-rays generated by X-ray apparatus DRON UM-2 with power supply PUR5/50 and an X-ray tube BSV-23 and a copper anode (Physics Department at Moscow State University, Russia) set to perform at U_A_ = 26 kV. During irradiation, the tube current was 30 mA, and the ambient temperature was 20 °C.

**Figure 3:**
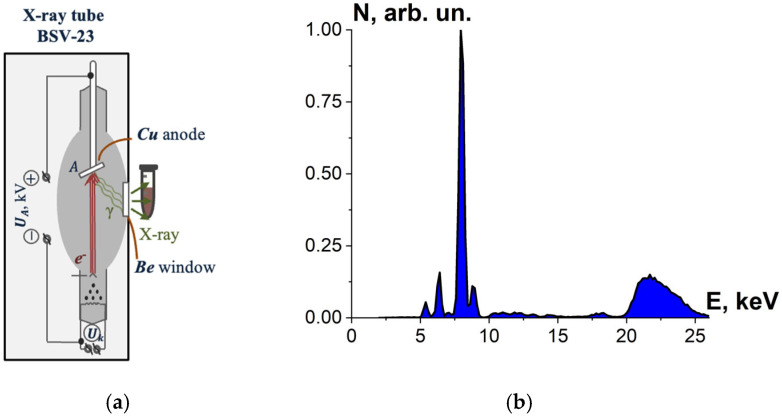
(**a**) X-ray irradiation method using X-ray apparatus DRON UM-2 with an X-ray tube BSV-23 and a copper anode; (**b**) X-ray energy spectrum.

The tubes with beef homogenate were placed vertically close to the beryllium window of the X-ray tube for irradiation with bremsstrahlung photons (Figure 3a), whose spectrum passing the beryllium window is shown in Figure 3b. The thickness of the homogenate layer was (7.0 ± 0.5) mm; irradiation of the beef homogenate was performed from two opposite sides. Six Eppendorf cylindrical tubes with beef homogenate were irradiated for each irradiation session.

**Figure 4:**
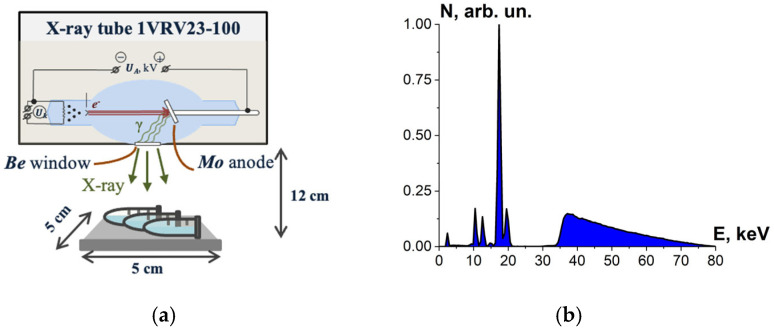
(**a**) X-ray irradiation method using X-ray apparatus RAP-100 with a 1VRV23-100 X-ray tube and a molybdenum anode; (**b**) X-ray energy spectrum.

Bovine serum albumin suspensions were irradiated with X-rays generated by X-ray apparatus RAP-100 (Burnasyan SRC-FMBC FMBA, Moscow, Russia) with a 1VRV23-100 X-ray tube and a molybdenum anode set to perform at U_A_ = 80 kV. During irradiation, the tube current was 10 mA and the ambient temperature was 20 °C. Each irradiation session involved three BSA suspensions in Eppendorf tubes placed 12 cm away from the beryllium window. The thickness of the albumin suspension layer was (2.0 ± 0.5) mm, and the samples were irradiated from one side (Figure 4a). The bremsstrahlung irradiation spectrum is shown in Figure 4b.

#### 2.1.5. Dosimetry Control

A ferrous sulfate (Fricke) dosimeter was used to estimate the dose absorbed by the samples during E-beam and X-ray irradiation. The irradiation of the Fricke dosimeter fully corresponded to the E-beam irradiation method for beef homogenate, BSA suspension in 0.5 mL Eppendorf tubes and for beef pieces in Petri dishes, as well as to X-ray irradiation method for beef homogenate and BSA suspension in 0.5 mL Eppendorf tubes.

The beef homogenate and beef pieces were irradiated with doses ranging from 250 Gy to 5000 Gy; albumin suspensions were irradiated with doses ranging from 150 Gy to 8000 Gy. According to the dose rates measured using the Fricke dosimeter, the dose rate was *P*_e_ = (2.0 ± 0.1) Gy/s for 1 MeV E-beam irradiation *P*_x_ = (1.9 ± 0.5) Gy/s for 26 keV X-ray irradiation generated by the X-ray apparatus DRON UM-2 and *P*_x_ = (2.3 ± 0.2) Gy/s for 80 keV X-ray irradiation generated by the X-ray apparatus RAP-100. As the dose rates for the electron and X-ray irradiation are close in value, it is possible to compare the effect of E-beam and X-rays with the same doses on proteins and microorganisms in beef samples.

#### 2.1.6. GEANT 4 Computer Simulation to Determine Dose Uniformity and Linear Energy Transfer in Irradiated Sample

The GEANT4 toolkit based on Monte Carlo method was used to control dose uniformity in beef pieces, beef homogenate and BSA suspensions during irradiation with accelerated electrons and X-rays.

To determine the absorbed dose uniformity distribution in the beef homogenate and the BSA suspensions irradiated with 1 MeV E-beam and in the BSA suspensions irradiated with 80 keV X-rays, the beef homogenate and the BSA suspensions were simulated as 37 mm × 7 mm × 2 mm parallelepiped water phantoms corresponding to the beef homogenate and the albumin suspension. The beef pieces irradiated with 1 MeV E-beam were simulated as 20 mm x 20 mm x 6 mm parallelepiped water phantoms. To determine the dose uniformity in the beef homogenate during 26 keV X-ray irradiation, the beef homogenate was simulated as a 7 mm cubic water phantom.

Each simulation of E-beam irradiation method was performed using 10^6^ electrons, with the energy spectrum shown in Figure 2b. Each simulation of X-ray irradiation was performed using 10^8^ photons, having two different energy spectra (Figure 3b and Figure 4b) depending on the type of the X-ray apparatus used for irradiation method. Totally, four computer simulations were performed for each type of phantom.

To calculate depth dose distributions and LET values in BSA suspensions during E-beam irradiation and X-ray irradiation, a 37 mm × 7 mm × 2 mm water parallelepiped and a 7 mm water cube were divided by thickness into twenty and seventy 0.1 mm layers for the parallelepiped and the water cube, respectively. The energy dE absorbed by each layer was estimated during computer simulation, and then the dose D absorbed by each layer was determined using the following formula:

(1) $$D_{layer}=\frac{{dE}_{layer}}{{dm}_{layer}},$$

where dm*_layer_* is the mass of the layer.

The average value of LET $\bar{L_{D}}$ over the absorbed energy was calculated by the formula:

(2) $$\bar{L_{D}}=\sum_{i=1}^{N}\frac{∆E_{i}}{∆l_{i}}w_{i},$$

where ∆*E_i_* is the energy released by the *i*-th electron in the sample layer, ∆*l_i_* is the length of the track of the *i*-th electron among all N electrons in the sample layer, *w_i_* is the weight factor determined by the formula:

(3) $$w_{i}=\left\{\begin{array}{c} \frac{{∆E}_{i}}{\sum_{j=1}^{N}{∆E}_{j}}\\ \frac{{∆l}_{i}}{\sum_{j=1}^{N}{∆l}_{j}} \end{array}\right..$$

#### 2.1.7. Microbiological Analysis of Beef Homogenate

For determining the quantity of viable cells, the beef homogenate irradiated with different doses was diluted with a 0.9% saline solution at ratios of 1:2, 1:10, 1:100, 1:1000 and 1:10,000 to obtain isolated cell colonies, expressed as the number of colony-forming units per gram (CFU/g). After that, 0.1 mL of the homogenate suspension was put in the nutrient medium, composed of 20.0 mg/L agar, 15.0 mg/L pancreatic casein hydrolysate, 5.0 mg/L yeast extract, 2.5 mg/L NaCl, 5.0 mg/L D-glucose, 0.5 mg/L sodium thioglycolate, 0.8 mg/L sodium carbonic acid, and 0.75 mg/L cysteine hydrochloride. All measurements and seeding were carried out under sterile conditions at 20 °C.

#### 2.1.8. Spectrophotometry Method to Determine the Concentration of Myoglobin Derivatives in Beef

To determine the metmyoglobin level, each beef piece was placed in a 5 mL 0.01M phosphate buffer solution mixed with 0.137 mol/L NaCl, after which the solution turned pink as myoglobin was released. After 15 min passed, a 1.5 mL supernatant solution was put into 2 mL Eppendorf tubes to be centrifuged using a Universal 320 centrifuge (Andreas Hettich GmbH & Co. KG, Kirchlengern, Germany) at 3500 rpm for 5 min. The solutions were subjected to spectrophotometry analysis at wavelengths ranging from 190 nm to 1100 nm using a spectrophotometer UV-3000 (TM ECOVIEW, Moscow, Russia) [47].

The resultant spectra of the solutions were mathematically processed using Origin Pro 2019 (OriginLab Corporation, Northampton, MA, USA) to determine myoglobin derivative concentrations. For this purpose, the spectra of the solutions were approximated by the formula:

(4) $$S_{k}{\left(\lambda_{k}\right)}_{theory}=\varepsilon_{Mb,l}C_{Mb}L+\varepsilon_{{MbO}_{2},l}C_{{MbO}_{2}}L+\varepsilon_{MetMb,l}C_{MetMb}L+\frac{G}{\lambda^{4}}+F$$

where *k* is the wavelength number; ε_MetMb,l_(*λ_k_*), ε_Mb,l_(*λ_k_*) and ε_MbO2,l_(*λ_k_*) are molar absorption coefficients of metmyoglobin, deoxymyoglobin and oxymyoglobin, which are close to molar absorption coefficients of hemoglobin derivatives [48]; *C*_MetMb_, *C*_Mb_ and *C*_MbO2_ are relative concentrations of metmyoglobin, deoxymyoglobin and oxymyoglobin, respectively, in relation to the total concentration of metmyoglobin, deoxymyoglobin and oxymyoglobin concentrations; *L* = 1 cm is the thickness of the solution layer; *G*, *F* are scattering coefficients [48,49].

#### 2.1.9. HPLC-MS Method to Assess the Potential Damage to Native Structure of Bovine Serum Albumin

The essence of the quantitative assessment of the potential damage to native structure of bovine serum albumin is to establish the presence of active form of bovine serum albumin (BSA) in 0.9% saline solution irradiated with electron beam, with the doses ranging from 150 Gy to 8000 Gy. This wide dose range was selected to trace and plot the relationship between the irradiation dose and the extent of the damage to protein native structure. Since an increase in irradiation dose causes more amide bonds in proteins to break, tracing active form of bovine serum albumin allows one to estimate the extent of the damage to the proteins—the source of nutrients—in order to inform the optimization function.

**Figure 5:**
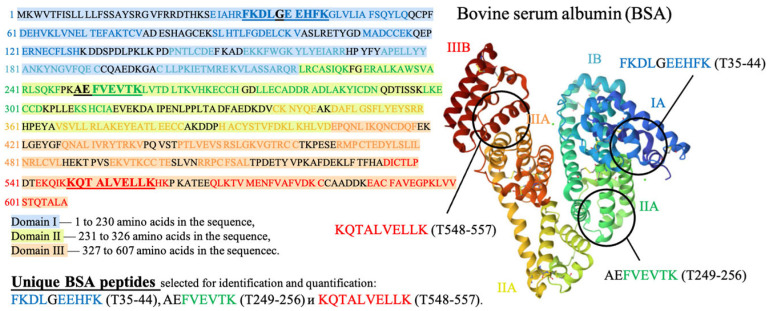
Amino acid sequence of bovine serum albumin (BSA) molecule.

Three peptides from albumin amino acid sequence, such as FKDLGEEHFK (T35-44), AEFVEVTK (T249-256) and KQTALVELLK (T548-557), which are present in one of the three different BSA domains (Figure 5), were selected to assess the potential damage to albumin protein native structure. The presence of all three selected peptides from the three different domains of the albumin chain in saline solution is a clear sign that no damage to albumin structure is detected using HPLC-MS method. A detailed description of the selection of peptides for estimation of BSA content in irradiated and non-irradiated BSA suspensions is provided in our previous paper [34].

Structural integrity of the native albumin form was analyzed on SMART Digest Trypsin Kit (Thermo Fisher Scientific, cat. No. 60109-101, Waltham, MA, USA) using the standard sample BSA fraction V (Bioloclot Gmbh, cat. No. 61171334, Aidenbach, Germany), formic acid, 95% (Sigma-Aldrich, cat. No. F0507, St. Louis, MO, USA), acetonitrile (Panreac, Barcelona, Spain), Amicon Ultra Centrifugal filters with molecular weight cut-off of 10 kDa (Amicon, No. Z677108, Darmstadt, Germany) and 30 kDa (Amicon, cat. No. UFC503024, Millipore, Carrigtwohill, Ireland), sodium chloride, 99% (Sigma-Aldrich, cat. No. S9888, USA), ammonium bicarbonate, 99% (Sigma-Aldrich, cat. No. A6141, USA), and deionized water after Milli-Q purification (Millipore, Temecula, CA, USA).

Peptide identification and quantification were performed using Ultimate 3000 RSLC liquid chromatograph (Thermo Fisher Scientific, Waltham, MA, USA) with automated sample input and Orbitrap Fusion Lumos high-resolution mass-selective tandem analyzer (Thermo Fisher Scientific, USA) with ion source and electrospray ionization. Selected BSA peptides were isolated using a 100 mm × 2.1 mm Zorbax 300 SB—C18 column with Ø 3.5 μm grain sorbent (Agilent, Santa Clara, CA, USA ). Chromatogram data were processed using Xcalibur software package version 4.2 (Thermo Fisher Scientific, Waltham, MA, USA). Before the solutions were centrifuged using MPW-352R centrifuge (MPW Med. Instruments, Warsaw, Poland), the temperature of the solutions required for enzymatic hydrolysis was maintained using MAXQ 4450 benchtop orbital shaker (Thermo Fisher Scientific, Waltham, MA, USA).

To identify and quantify the concentration of the selected albumin peptides, 210 µL of 1M NH_4_HCO_3_ was added to 30 µL of 0.9% NaCl aqueous solution, placed in a 0.5 mL Amicon centrifuge filter with a 30 kDa mass cut-off and centrifuged for 15 min at 10,000 rpm. After that, 50 µL protein concentrate from the filter was centrifuged for 3 min at 1000 rpm and put into a 2 mL Eppendorf tube. Next, 12 μL of 1M NH_4_HCO_3_ solution and 90 μL of buffer solution from the trypsin hydrolysis kit were added to the protein concentrate. After stirring the resulting mixture, 3 µL of 1 mg/mL trypsin solution was added to the mixture and stirred in a vortex mixer. The mixture was then incubated in a thermostat at 70 °C for 2 h and stirred again in the vortex mixer before loading into a 0.5 mL Amicon centrifuge filter with a 10 kDa mass cut-off after for ten-minute centrifugation at 2000 rpm. After the preparation, the mixture was placed in a Micro-Flask for HPLC-MS analysis.

To obtain the chromatograms during HPLC-MS/MS analysis, we used an ion source with electrospray emission. The resolution of the mass analyzer was not less than 30,000, and the error in determining the *m*/*z* value did not exceed 3,000,000^−1^. The temperature of the transfer capillary was 300 °C, the voltage on the atomizing capillary was 3500 V, and the pressure of the gas for atomization of the mobile phase in the ion source was 420 kPa. The components of the studied mixture were separated using gradient elution with a mobile phase flow rate 0.30 mL/min and a column thermostat temperature was 40 °C. The mobile phase A was 0.1% of the volume of HCOOH in water; mobile phase B was acetonitrile. Gradient elution program was as follows: 0–3 min: 95% A; 3–35 min: 5–35 min: 40% B; 35–40 min: 40–80% B; 40–44 min: 80% B; 44–50 min: 95% A. The volume of the injected sample was 10 μL.

### 2.2. Potato Pre-Planting Irradiation Methodology to Find the Optimal Dose Range

#### 2.2.1. Objects of This Study

Research was conducted on naturally infected Lina seed tubers, with a starch content of 14–20%, resistant to cancer, late blight and macrosporiosis, and mosaic viruses [50], grown at Siberian Federal Scientific Centre of Agrobiotechnologies under the Russian Academy of Science. The purpose of the research was to inhibit *Rhizoctonia solani Kühn* (*R. solani*) in harvested potatoes.

Identical Ø (40 ± 5) mm ellipsoid tubers with a depth of sclerotia penetration of around 2 mm were selected for the experiment to ensure the uniformity of data obtained during this study. Eighty seed tubers were monitored after irradiation, with ten doses ranging from 20 Gy to 200 Gy to study the impact of a wider dose range applied to seed potatoes before planting on the potato yield and phytosanitary condition.

Mycelium cells of *R. solani* put in potato-dextrose agar (PDA), isolated from diseased leaves, stems, and seeds, were irradiated in Ø 37 mm Petri dishes to compare the impact of accelerated electrons on the suppression of pure cultures of phytopathogens and phytopathogens, which can be found in potato tubers.

#### 2.2.2. Research Stages

**Figure 6:**
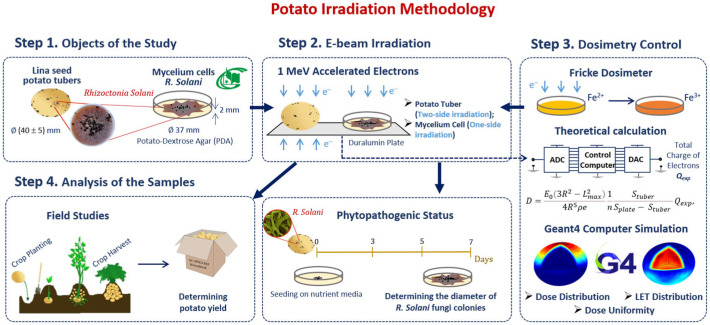
Research stages to determine optimal dose range for potato pre-planting irradiation.

Our methodology consisted of preparation and irradiation of seed potatoes, dosimetry control and a two-phase analysis of the harvested potato tubers grown from irradiated and non-irradiated seed potatoes (Figure 6). Seed potatoes naturally infected with *R. solani* were irradiated with 1 MeV E-beam (Figure 6 Step 2) and planted in an open field (Figure 6 Step 4). The doses absorbed by the seed potato tubers were calculated using an analytical formula taking into account the electron charge absorbed by the potato tubers during E-beam irradiation. To assess the impact of 1 MeV accelerated electrons on the *R. solani* suppression efficiency, GEANT 4 computer simulation was carried out for estimating the dose distribution over the volume of seed potatoes (Figure 6 Step 3). Harvested tubers were examined to determine the ratio of the tubers infected with *Rhizoctonia sclerotia* and the total yield. The ratio of *Rhizoctonia* infected tubers and the total yield were used to determine the optimal dose range for pre-planting E-beam irradiation of seed potatoes (Figure 6 step 4).

To study the impact of irradiation on the suppression of pure cultures of phytopathogens, *R. solani* mycelium cells were irradiated with 1 MeV accelerated electrons within doses from 100 Gy to 10,000 Gy (Figure 6 step 2). Fricke dosimetry was used to estimate the dose absorbed by the mycelium mat (Figure 6 step 3). Five days after irradiation, the diameters of fungi colonies grown from the cells cut from the non-irradiated and irradiated mycelium mat were measured to estimate the phytopathogen suppression efficiency (Figure 6 step 4), which was compared to the suppression efficiency of *Rhizoctonia sclerotia* located on the surface of the harvested potato tubers grown from the irradiated and non-irradiated seed potato tubers.

#### 2.2.3. Low-Energy Electron Beam Irradiation of Seed Potato Tubers

The purpose of this stage of the research is to find the optimal doses for pre-planting irradiation of seed potato tubers, which could suppress phytopathogens on the new harvest tubers with a minimal negative impact on the crop yield. The choice of the 1 MeV electron beam irradiation method for pre-planting irradiation of seed potatoes is determined by the fact that 1 MeV electron penetration depth corresponds to the depth at which phytopathogens can be found in potato tubers. To ensure uniform surface irradiation, Ø (40 ± 5) mm Lina seed potato tubers infected with *R. solani* were irradiated with 1 MeV electron beam, with the energy spectrum shown in Figure 6 from two opposite sides.

#### 2.2.4. Estimation of the Dose Absorbed by the Tubers During Irradiation

The dose absorbed by the tubers during irradiation was calculated by the following formula [35]:

(5) $$D=\frac{E_{0}\left(3R^{2}-L_{max}^{2}\right)}{4\pi R^{5}\rho e}\frac{1}{n}\frac{S_{tuber}}{S_{plate}-S_{tuber}}Q_{exp},$$

where *E*_0_ = 1 MeV is the effective electron beam energy; *R* = 30 mm is the average radius of the potato tuber; *S_plate_* = 192 cm^2^ is the size of duralumin plate; *S_tuber_* = 28.3 cm^2^ is the shadow of one tuber; *n* = 6 is the number of tubers located simultaneously on duralumin plate during each irradiation session; *L*_max_ = 5.5 mm is the maximum penetration depth of 1 MeV electrons in the tuber; *e* is the electron charge. The value *Q_exp_* is the charge absorbed by the area of the duralumin *S_plate_* free from the tubers and measured using the analog-to-digital converter (OOO Industrial Association “Oven”, Russia). As shown in Table 1, the margin of error in determining the charge *Q_exp_* absorbed by the plate does not exceed 2%. Since the density of the tuber is close to that of water, it is assumed that in Formula (5), the potato density is *ρ* = 1 g/cm^3^. The calculations show that the tubers were irradiated with doses ranging from 20 Gy to 200 Gy.

#### 2.2.5. Low-Energy Electron Beam Irradiation of Phytopathogen

Pure cultures of *R. solani* put in Ø 37 mm Petri dishes with PDA were irradiated with electrons at doses of 100, 1000 and 10,000 Gy. The number of Petri dishes per irradiation dose was *n* = 3.

#### 2.2.6. Estimation of the Dose Absorbed by Phytopathogens During Irradiation

Fricke dosimeter was used for estimating the irradiation dose absorbed by *R. solani* mycelium cells. A 4.5 mL Fricke dosimetry solution was put in Ø 37 mm Petri dishes to estimate the dose absorbed by the phytopathogens *R. solani*. The thickness of dosimetry solution was (4.0 ± 0.1) mm. The irradiation method for Fricke dosimeter fully corresponded to the irradiation method for phytopathogens *R. solani*. The dose rate for electron irradiation was *P*_e_ = (1.2 ± 0.1) Gy/s.

#### 2.2.7. Potato Productivity Analysis

Field studies were carried out under soil and climatic conditions typical for the forest-steppe zone of Western Siberia. After planting the tubers, the research team recorded the number of days required for the onset of germination, budding and flowering phases of plants. Further in the research, after estimating the yield, the team performed fractional analysis of tubers and assessed their phytosanitary status.

#### 2.2.8. Analysis of Phytosanitary Condition of New Crop Tubers

After pre-planting irradiation, seed potato tubers were planted at the experimental field OS Elitnaya in proximity to Novosibirsk in medium-thick leached chernozem. Immediately after harvesting, the 1/10, 1/4 and 1/2 of the surface of the potato tubers were carefully examined to determine the ratio of the tubers infected with *Rhizoctonia sclerotia* in the yield. The data obtained were statistically analyzed using SENECOD mathematical toolkit [51].

#### 2.2.9. Estimation of the Diameters of Phytopathogen Colonies

After irradiation, 5 mm diameter disks were cut from the *R. solani* mycelium mats with a cork drill and put into Ø 37 mm sterile plastic Petri dishes with Czapek’s Agar supplemented with streptomycin and chloramphenicol for further incubation at 25 °C for 7 days after irradiation. After five days of incubation, the diameter of fungi colonies was registered to estimate the phytopathogen suppression efficiency.

## 3. Results and Discussion

### 3.1. Factors Influencing Food Irradiation Efficiency

**Figure 7:**
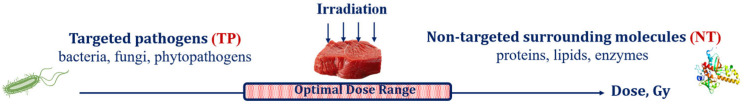
Irradiated food product as a combination of targeted pathogens and non-targeted surrounding molecules.

Food irradiation, aimed at destroying targeted pathogens (TPs), such as bacteria, viruses, phytopathogens and fungi, inevitably causes damage to surrounding molecules, such as proteins, lipids, carbohydrates and enzymes, further referred to as non-targeted (NT) biological structures, to the extent, marked by ε, that is determined by the physical properties of irradiation as well as biological and chemical features of the irradiated food product. Considering that irradiation aimed at pathogen suppression inevitably damages the surrounding molecules in the food product, the optimal irradiation dose range should be able to maximize the suppression of pathogens while minimizing the negative impact to the surrounding molecules (Figure 7). It is also important to note that the optimal dose range for each food category depends on both the physical factors of interaction between irradiation and the substance of the product and biological factors responsible for the individual properties of pathogens and the surrounding molecules in the irradiated product.

#### 3.1.1. Factor K_1_ and Irradiation Dose Uniformity

**Figure 8:**
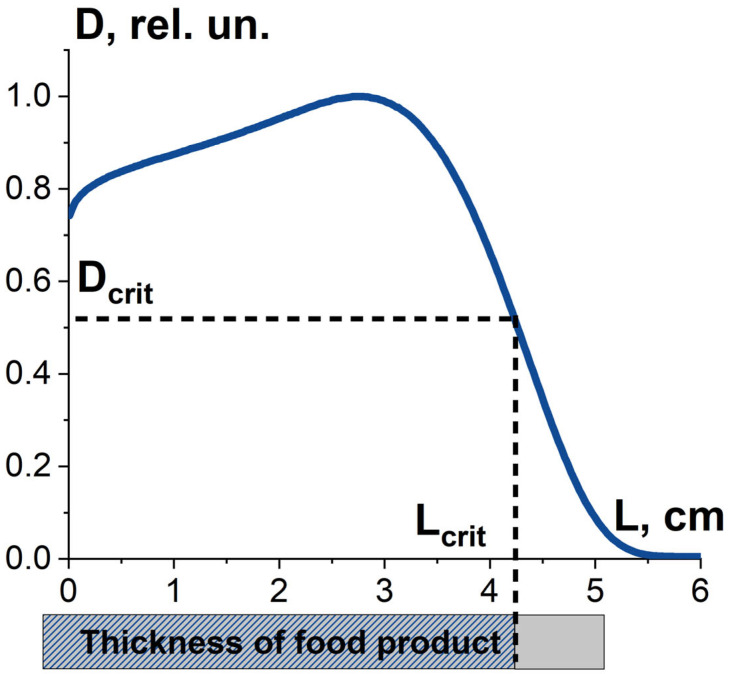
Factor K_1_ is the combination the absorbed dose uniformity in food product irradiated with 10 MeV electrons and the dose required to damage bio-targets to a given level.

Efficient suppression of pathogens in the irradiated food product is determined by the dose uniformity U, which is represented as follows:

(6) $$U=\frac{D_{min}}{D_{max}},$$

where *D_min_*, *D_max_* are minimum and maximum absorbed dose over the volume of the food product. Along with dose uniformity, the critical dose required to damage pathogens to a given level determines the thickness L_crit_ and volume of food product, in which pathogens are suppressed as required. Therefore, the combination of the dose uniformity U and the dose D_crit_ determines the value K_1_, which is the ratio of the suppressed pathogens to all pathogens in the food product exposed to radiation. It should be noted that the value K_1_ is determined not only by the irradiation parameters, such as irradiation type, energy spectrum and irradiation fluence, but also by the type of pathogens (Figure 8).

#### 3.1.2. Factor K_2_ and Probability of Irradiation Hitting Pathogens and Surrounding Molecules

**Figure 9:**
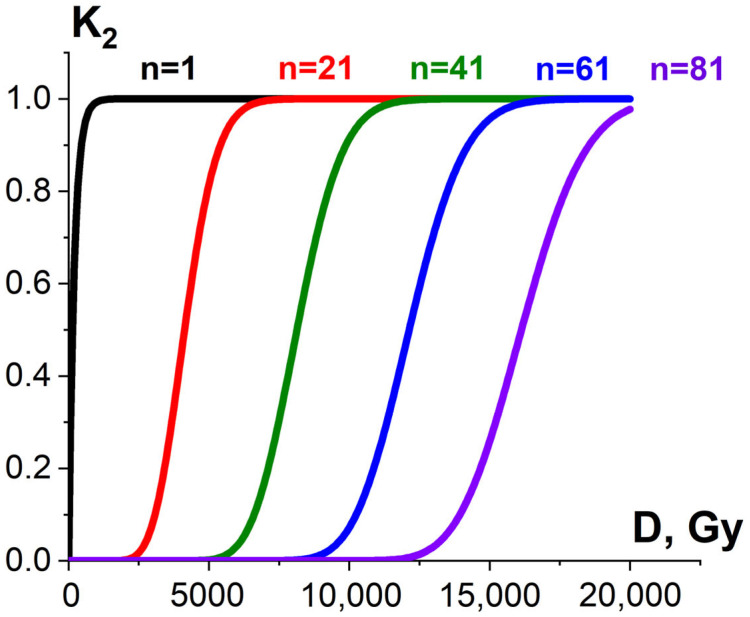
Exponential (black curve) and sigmoidal (red, green, blue and violet curves) dose dependencies of homogeneous damaged bio-targets K_2_ on the irradiation dose.

Since irradiation hitting pathogens or surrounding molecules is a random occurrence, both the pathogen suppression efficiency ε^TP^ and the extent at which the surrounding molecules are damaged ε^NT^ are determined by the number of ionization events required to damage pathogens and surrounding molecules. According to target theory in radiobiology [52], if one ionization event *n* = 1 is sufficient to damage the targeted or non-targeted biological structures of the same radiosensitivity, then the ratio of damaged pathogens or surrounding molecules K_2_ exponentially depends on the dose and can be expressed as follows:

K_2_(D) = 1 − e^−*αD*^, (7)

where *α* (Gy^−1^) is determined by linear energy transfer (LET), linear dimensions of pathogens or surrounding molecules as well as the individual radiosensitivity of pathogens or surrounding molecules. If more than one ionization act is required to damage pathogens or surrounding molecules, the dependency of K_2_ on the irradiation dose is sigmoidal [52] (Figure 9).

#### 3.1.3. Factor K_3_ and Heterogenic Radiosensitivity of Pathogens and Surrounding Molecules

**Figure 10:**
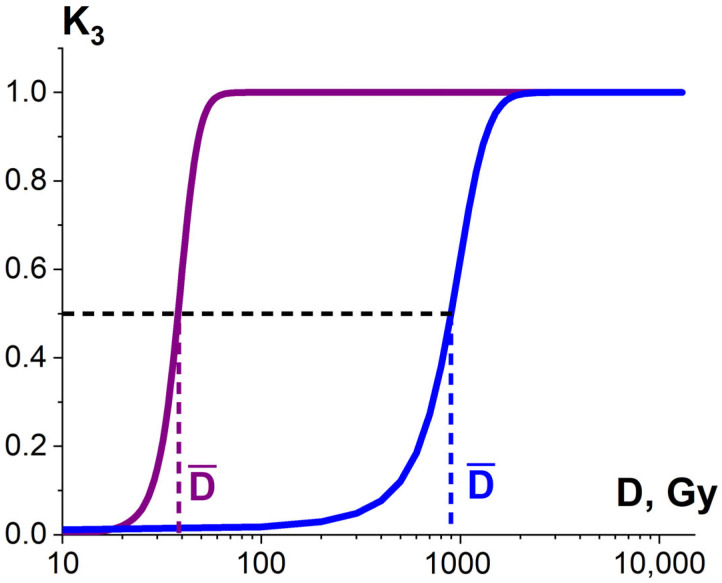
Dependencies of the ratio of the damaged bio-targets *K*_3_ in two different statistical ensembles on the irradiation dose.

Considering that the targeted pathogens or non-targeted surrounding molecules can have diverse radiosensitivity, the number of ionization acts *n* required to damage pathogens or surrounding molecules differs depending on the arrangement of pathogen clusters and the biochemical composition of the food product. Therefore, the ratio of the damaged pathogens or surrounding molecules K_3_ is described by the sigmoidal function (Figure 10), expressed as follows:

(8) $$K_{3}\left(N\right)=\frac{1}{1+e^{-\frac{\left(N-\bar{N}\right)}{\delta }}},$$

where $\bar{N}$ is the number of ionization acts leading to the damage to 50% of pathogens or surrounding molecules, and δ is the width of the transition region of the function (8).

Therefore, the factors K_1_, K_2_, K_3_ are determined by different mechanisms behind the damage to biological structures and depend on the applied irradiation dose (Figure 8, Figure 9 and Figure 10). The degree of damage incurred by targeted pathogens ε^TP^ and non-targeted surrounding molecules ε^NT^ is the function of the values K_1_, K_2_, K_3_, and the dose dependency ε(D) for each biological structure is determined by the physical properties of irradiation and food product, as well as the number of ionization events required to damage the structures, which varies depending on the type of structure.

### 3.2. The Influence of K_1_, K_2_, K_3_ Factors on Pathogen Inactivation and Damage to Proteins

A series of experiments conducted by our team showed that the pathogen inactivation efficiency is greatly affected by factor K_1_, which is the combination of irradiation dose uniformity and the dose D_crit_ required to damage pathogens to a given level. Another factor affecting pathogen inactivation efficiency is K_2_, which is determined by the probability of damage to pathogens caused by irradiation. One more factor K_3_ that has a role to play in pathogen inactivation is the heterogeneity of radiobiological sensitivity. Therefore, pathogen suppression efficiency ε^TP^ and the extent of the damage to surrounding molecules ε^NT^ are the functions of three factors, each having its own dose dependency:

(9) $$\varepsilon (D)=F\left[K_{1}=f_{1}(D),K_{2}=f_{2}(D),K_{3}=f_{3}(D)\right].$$

As our experiments show, the dependencies of suppression efficiency ε^TP^ and the extent of the damage to surrounding molecules ε^NT^ on the irradiation dose can be either exponential or sigmoidal. It should be noted that exponential dependencies are typical for smaller biological structures, such as bacteria and proteins [23,34], that tend to have similar radiosensitivity and whose factor K_3_ tends towards 1. Summarizing all the formulas for each of three factors (6)–(8), the exponential dose dependency ε(D) can be expressed as follows:

(10) $$\varepsilon (D)=K_{1}(D)\cdot K_{2}(D)=K_{1}(D)\cdot \left(1-e^{-\alpha D}\right).$$

The sigmoidal dependencies ε(D), which are represented as

(11) $$\varepsilon (D)=K_{1}(D)\cdot K_{3}(D)=K_{1}(D)\cdot \frac{1}{1+e^{-\frac{\left(N-\bar{N}\right)}{\delta }}},$$

can be observed when biological structures are easily accessible for irradiation, which makes the probability of irradiation hitting biological structures relatively high. For example, the suppression efficiency ε^TP^ of phytopathogens in seed potatoes tends to be suppressed by irradiation immediately, since they can be found in the surface layers of potato tubers.

#### 3.2.1. Factor K_1_ and Pathogen Suppression Efficiency

To assess the degree to which dose uniformity influences the pathogen inactivation efficiency, we used irradiation doses ranging from 250 Gy to 3000 Gy and a dose rate of 2 Gy/s to compare different irradiation types in terms of their pathogen inactivation power. Beef homogenate containing (1.0 ± 0.2) × 10^4^ CFU/g mesophilic aerobic and facultative–anaerobic microorganisms was irradiated with 1 MeV electrons using the electron accelerator UELR-1-25-T-001 and with X-rays generated by a DRON UM-26 X-ray apparatus with a BSV 23 X-ray tube. The thickness of the beef homogenate, which determines the dose uniformity, was (2.0 ± 0.5) mm during one-side electron beam irradiation and (7.0 ± 0.5) mm during two-side X-ray irradiation.

The microorganism suppression efficiency $\varepsilon_{exp}^{TM}$ was estimated during the experiment as follows:

(12) $$\varepsilon_{exp}^{TM}=1-\frac{\bar{N_{irr}}}{\bar{N_{ref}}}$$

where $\bar{N_{irr}}$ and $\bar{N_{ref}}$ are the average numbers of viable cells in irradiated and non-irradiated beef homogenate samples, respectively.

**Figure 11:**
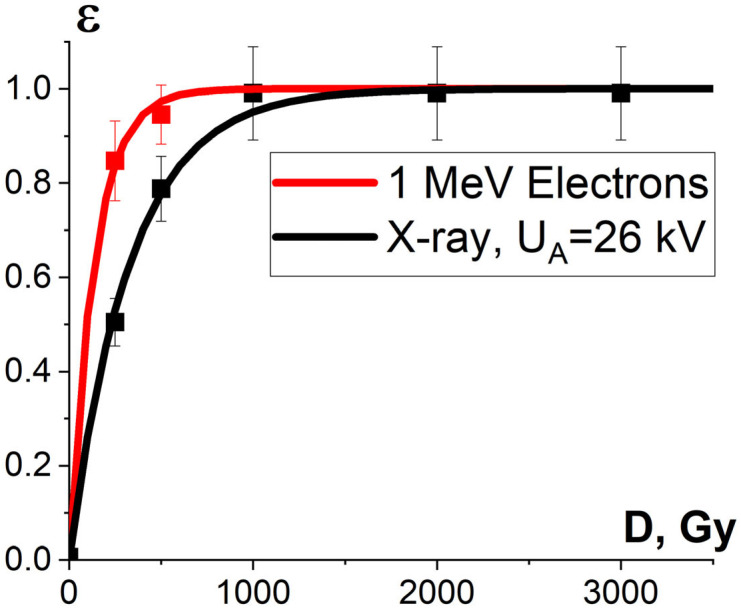
Experimental dependencies of the number of viable cells, shown by squares, and calculated dependencies of microorganism inactivation efficiency ε^TM^(D), shown as lines, on the irradiation dose in beef homogenate irradiated with electron beam (red curve) and X-ray irradiation (black curve). The approximation of experimental data according to Formula (10) was performed with the following parameters: K_1_ = 0.99 ± 0.01, α = (0.0073 ± 0.0003) Gy^−1^, R^2^ = 0.99 for electrons; K_1_ = 0.96 ± 0.03, α = (0.0031 ± 0.0001) Gy^−1^, R^2^ = 0.99 for X-ray radiation.

Figure 11 shows the dose dependencies of microorganism inactivation efficiency ε^TM^(D) in the beef homogenate irradiated with accelerated electrons and X-rays. As can be seen from Figure 11, the inactivation efficiency of microorganisms in the beef homogenate grows exponentially with an increase in the irradiation dose for both types of irradiation, which can be expressed by Formula (10). In the case of electron beam irradiation, the dose D_10_, which reduces the number of viable cells in the beef homogenate ten-times, is (316 ± 14) Gy, while for X-rays, this parameter amounts to (761 ± 30) Gy. Therefore, when the beef homogenate was irradiated with electrons, a lower dose was required to suppress the same amount of bacteria compared to X-ray irradiation. When it comes to thermal processing, the same effect of the suppression of bacteria commonly found in beef is achieved at 60 °C [53], which changes the nutrient value, organoleptic properties and the texture of the product. Compared to thermal processing, food irradiation with doses ranging from 250 Gy to 3000 Gy only warmed the beef homogenate to a fraction of a degree, without causing a negative impact on the quality of beef. The number of viable cells from irradiated and non-irradiated beef homogenate is summarized in Appendix A in Table A1.

One-way ANOVA analysis, applied to compare the impact of accelerated electrons and X-rays on microorganisms, shows that in the dose range of 250–800 Gy, electron beam irradiation is able to suppress microorganisms at a level that is statistically higher (*p* ≤ 0.05) than that registered during X-ray irradiation. To explain the difference between the microorganism inactivation efficiency of accelerated electrons and X-rays, we performed the GEANT 4 computer simulation to calculate the depth dose distribution in water parallelepiped with a thickness of 7 mm for electrons and X-rays. The water parallelepiped simulating the beef homogenate during electron irradiation was hit by electrons from one side in line with the E-beam irradiation method, which had been used to irradiate the beef homogenate. The water parallelepiped simulating the beef homogenate during X-ray irradiation was exposed to bremsstrahlung photons generated by 26 keV electrons hitting the copper anode according to the physical and technical parameters of the DRON UM-26 X-ray apparatus with a BSV 23 X-ray tube set to perform at 26 kV. The simulation procedure involving X-ray irradiation of the water parallelepiped complies with the X-ray irradiation method, which had been used to irradiate the beef homogenate (Figure 3a).

**Figure 12:**
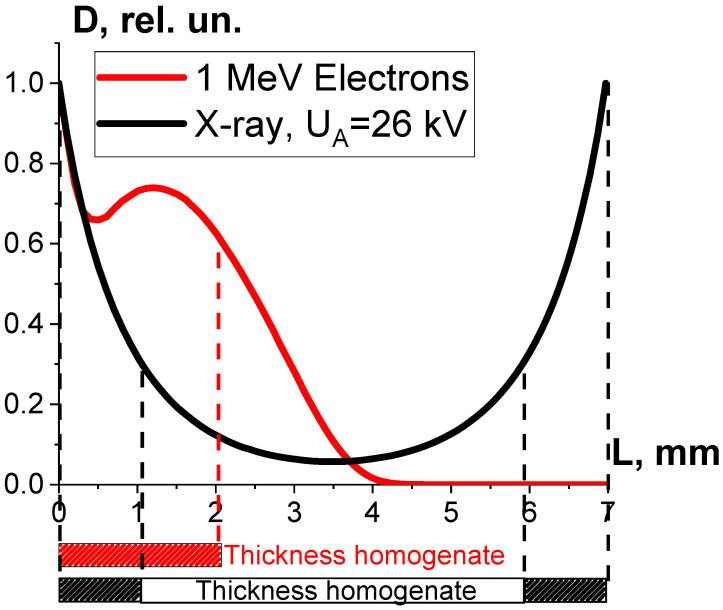
Depth dose distributions in a 7 mm thick water parallelepiped irradiated with 1 MeV accelerated electrons (red curve) and X-rays with the maximum energy of 26 keV (black curve).

As can be seen, while the dose uniformity U in the beef homogenate irradiated with accelerated electrons is 0.6, the dose uniformity in the X-rayed beef homogenate is 0.1 (Figure 12). According to Formula (10), the microorganism inactivation efficiency ε^TM^(D) depends on the factor K_1_, which is a combination of the dose uniformity U and D_crit_, the dose needed for the inactivation of microorganisms to a required level. The dose uniformity U in the beef homogenate irradiated with accelerated electrons is higher than that of X-ray irradiated beef, and the dose range between 250 Gy and 800 Gy showed a higher microorganism inactivation efficiency for accelerated electrons compared to X-ray irradiation. This experiment illustrates the fact that factor K_1_ has a significant impact on the microorganism inactivation efficiencies in the beef homogenate irradiated with the two radiation types within a dose range of 250–800 Gy.

#### 3.2.2. Factor K_3_ and Efficiency of Phytopathogen Suppression

**Figure 13:**
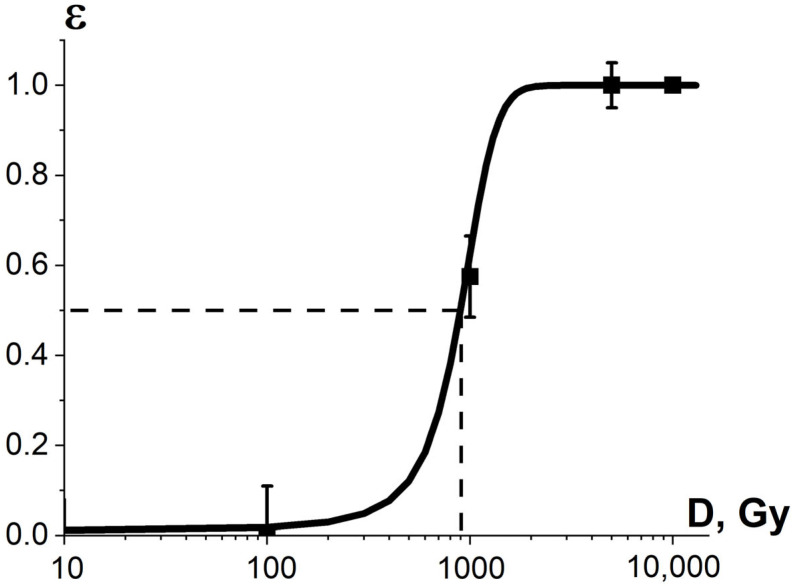
Experimental dependencies of the relative diameter of fungi colonies *R. solani* (squares) grown from mycelium mat sections and calculated dependencies of phytopathogen suppression efficiency shown as line on the irradiation dose. The approximation of experimental data according to Formula (11) was performed with the following parameters: $K_{1}=1,\;\bar{D}=(896\pm 12)$ Gy, *δ* = (208 ± 11) Gy, R^2^ = 0.99.

The 1 MeV electron beam irradiation of pure cultures of *R. solani* fungi revealed that the average diameter $\bar{d_{irr}}$ of fungi grown from irradiated mycelium cells, which were taken from different parts of fungi after irradiation, decreased with an increase in the irradiation dose. The phytopathogen suppression efficiency $\varepsilon_{exp}^{TM}$ was estimated during the experiment as follows:

(13) $$\varepsilon_{exp}^{TM}=1-\frac{\bar{d_{irr}}}{\bar{d_{ref}}},$$

where $\bar{d_{ref}}$ is the average diameter of fungi grown from non-irradiated mycelium cells. The data on $\bar{d_{irr}}$ and $\bar{d_{ref}}$ were received from three consecutive iterations of calculations of the diameter of fungi grown from irradiated and non-irradiated mycelium cells. The average diameters of fungi grown from irradiated and non-irradiated mycelium cells are summarized in Appendix A in Table A2. As can be seen from Figure 13, the experimental dependency $\varepsilon_{exp}^{TM}$(D) is sigmoidal and can be expressed using Formula (13).

The sigmoidal dose dependencies for phytopathogen suppression efficiency show that different fungi cells taken from different sections of the same mycelium mat require a different number of ionization events to damage the cells to a critical level, which depend on the condition and the life cycle of the cells. As the research suggests, phytopathogens, commonly found in crops, have variable radiobiological sensitivity, and the uniform E-beam irradiation of phytopathogens proved that the heterogeneity of radiobiological sensitivity K_3_ has a significant impact on the pathogen suppression efficiency in crops.

#### 3.2.3. Factor K_2_ and Damage to Protein Native Structure

Since the irradiation of a food product has an impact not only on the pathogens but also on the surrounding molecules, the factors K_1_, K_2_ and K_3_ influence the extent of the damage to the surrounding molecules. A series of experiments conducted by our team shows that the potential damage to the native structure of bovine serum albumin (BSA) after irradiation is greatly affected by the factor K_2_, which stands for the probability of irradiation hitting the peptides selected from the albumin native structure. Since the value K_2_ depends on the linear energy transfer (LET) and the linear dimensions of peptides, the irradiation of albumin suspension with different types of irradiation having different distributions of LET in the volume of the irradiated suspension suggests that K_2_ has a notable impact on the extent of the damage to peptides.

To assess the influence of factor K_2_ on the extent of the damage to albumin, we compared the influence of 1 MeV electrons generated by an electron accelerator UELR-1-25-T-001 and X-rays generated by X-ray apparatus RAP-100 with the X-ray tube 1VRV23-100 set to perform at 80 kV on the extent of the damage to the peptides from the BSA amino acid sequence. For that purpose, BSA in 0.9% saline solution with a concentration of 0.5 mg/mL was irradiated with doses ranging from 150 Gy to 8000 Gy and a dose rate of 2 Gy/s. The thickness of the albumin solution, which determines the dose uniformity, was (2.0 ± 0.5) mm for both one-side electron beam irradiation and one-side X-ray irradiation.

**Figure 14:**
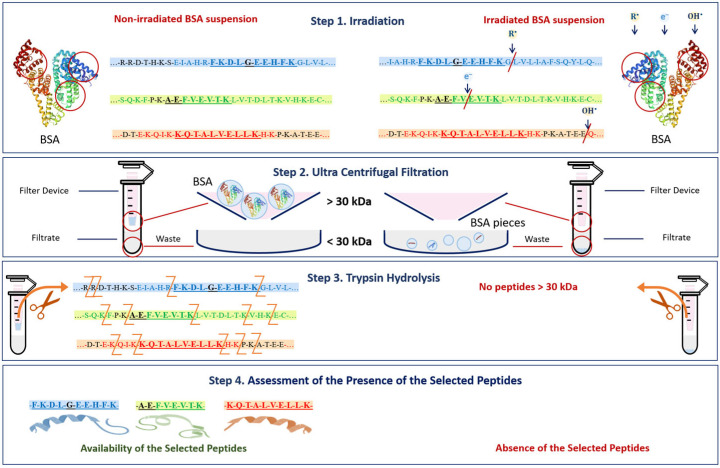
The mechanism of the change in BSA native structure after irradiation and the detection of these changes after trypsin hydrolysis.

The mechanism of the change in the BSA native structure after irradiation and the detection of these changes after trypsin hydrolysis are shown in Figure 14. Since the denaturation of proteins caused by the breaking of amide bonds between amino acids and the formation of low-molecular-weight peptides may occur as a result of irradiation (Figure 14 Step 1) [54], at the second stage of the research, it was necessary to ensure that bovine serum albumin was present in its native form. For this purpose, a 0.5 mL Amicon filter with a 30 kDa mass cut-off was used to eliminate low-molecular-weight protein degradation products (Figure 14 Step 2) that could give false-positive results during the identification of the selected peptides after trypsin hydrolysis (Figure 14 Step 3). The hydrolysate obtained during trypsin hydrolysis was analyzed using the HPLC-MS method to quantify the concentration of the selected BSA peptides, having *m*/*z* values of 417, 461 and 571 and present in three different domains of the BSA (Figure 14 Step 4). The content of the selected peptides in the irradiated BSA suspensions was normalized relative to the non-irradiated suspensions. The suggested method allows one to quantify the extent of the potential damage to the protein native structure after irradiation.

To experimentally assess the potential damage to the BSA native structure $\varepsilon_{exp}^{NT}$ after irradiation with accelerated electrons and X-ray irradiation at different doses, we calculated the amount of damaged peptides using the following formula:

(14) $$\varepsilon_{exp}^{NT}=1-\frac{\bar{C_{irr}}}{\bar{C_{ref}}},$$

where $\bar{C_{irr}}$ and $\bar{C_{ref}}$ are the concentrations of three selected peptides measured in irradiated and non-irradiated BSA suspensions, respectively. The results of the impact of accelerated electrons and X-rays on the content of the selected peptide having an *m*/*z* value of 461 in BSA suspensions irradiated with E-beam and X-rays at doses ranging from 150 Gy to 8000 Gy are presented in Appendix A in Table A3. The data on the concentration of the peptide were received from three consecutive iterations of peptide content estimation.

**Figure 15:**
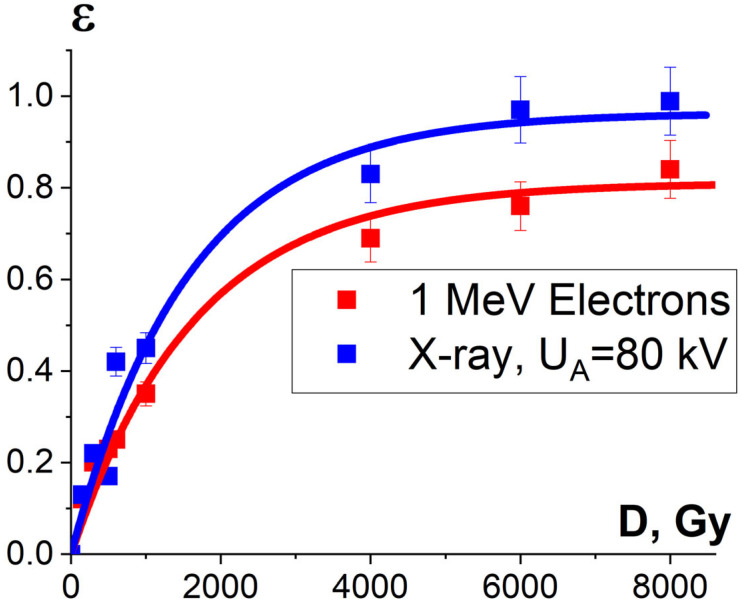
Dependencies of the damaged peptides from BSA sequence $\varepsilon_{exp}^{NT}(D)$, calculated using Formula (14) and shown by squares, and calculated dependencies of protein damage efficiency ε^NT^(D), shown as lines, on the irradiation dose absorbed by the BSA suspension during electron beam irradiation (red curve) and X-ray irradiation (blue curve). The approximation of experimental data according to Formula (10) was performed with the following parameters: K_1_ = 0.90 ± 0.01, α = (0.00060 ± 0.00006) Gy^−1^, R^2^ = 0.96 for electrons; K_1_ = 0.96 ± 0.04, α = (0.00064 ± 0.00009) Gy^−1^, R^2^ = 0.97 for X-ray radiation.

It was found that the relative concentration of the damaged peptides with the *m*/*z* values of 417, 461 and 571 increased exponentially with an increase in the irradiation dose and differed within 5% for three different peptides. Figure 15 shows the experimental dependencies of the relative concentration of one of three selected peptides $\varepsilon_{exp}^{NT}(D)$ with the *m*/*z* value of 461 on the irradiation dose created by accelerated electrons and X-rays and the dependencies ε^NT^(D) calculated using Formula (10).

**Figure 16:**
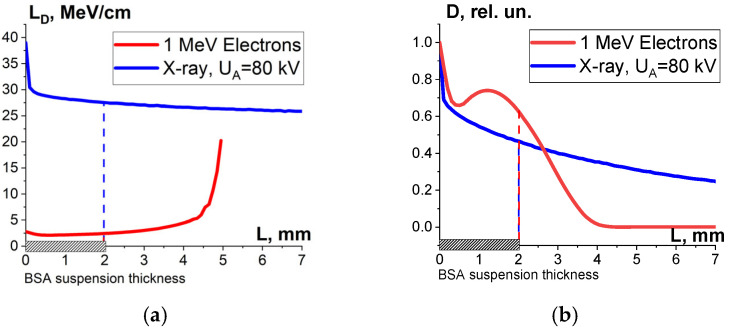
Result of the water parallelepiped being irradiated with 1 MeV electrons (red curves) and X-rays having the maximum energy spectrum of 80 keV (blue curves): (**a**) LET; (**b**) relative absorbed dose distributions.

One-way ANOVA analysis showed that in the dose range of 600–2000 Gy, the relative concentration of the peptide with an *m*/*z* value of 461 damaged by X-rays is statistically higher (*p* ≤ 0.05) than that for E-beam irradiation (Figure 15). To investigate the reason for a higher efficiency of X-rays with the maximum energy of 80 keV in the spectrum compared to 1 MeV accelerated electrons, we simulated LET for electrons and X-rays distributed in different layers of a 2 mm thick water parallelepiped, whose thickness corresponds to the thickness of the BSA suspension during irradiation (Figure 16). The LET values, averaged over the energy absorbed by different layers of the water parallelepiped, were calculated using Formula (2), taking into account the energy spectrum for the electron beam generated by the electron accelerator UELR-1-25-T-001 (Figure 2b) and the energy spectrum for X-rays (Figure 4b) generated by X-ray apparatus RAP-100. To trace the influence of dose uniformity factor K_1_, we estimated depth dose distributions in a 2 mm thick water parallelepiped irradiated with accelerated electrons and X-rays (Figure 16b). We found that for X-ray irradiation, the LET values in a 2 mm thick water layer vary from 27.5 MeV/cm to 38.3 MeV/cm, while for electrons, the LET values range from 2.4 MeV/cm to 2.6 MeV/cm (Figure 16a) at a dose uniformity of U = 0.62 ± 0.03 for electron beam irradiation and U = 0.43 ± 0.01 for X-ray irradiation (Figure 16b). Thus, it can be concluded that the efficiency of the damage to the BSA native structure ε^NT^ is higher when the BSA suspension is irradiated with X-rays in a dose range of 600–2000 Gy compared to that for accelerated electrons because the LET values of the 80 keV X-ray irradiated water parallelepiped are ten-times higher than the LET of the water parallelepiped irradiated with 1 MeV accelerated electrons. Since factor K_2_ depends on the LET values generated by irradiation in a food product, the efficiency of damage to the BSA native structure was largely determined by K_2_. Therefore, the method involving the quantitative assessment of potential damage to the native structure of BSA using trypsin hydrolysis allows one to compare the LET values for different irradiation types. The clear dose-dependent relationship between the concentration of selected peptides and the irradiation dose shows that the potential damage to the albumin native structure can serve as a reliable irradiation marker for foods containing a great amount of proteins.

The electron beam irradiation and X-ray irradiation of albumin suspensions and biological objects, such as beef, pure cultures of phytopathogen, confirm the significant influence of K_1_, K_2_ and K_3_ factors on the efficiency of pathogen inactivation and the rate of damage to the surrounding molecules. Exponential dose dependencies of bacteria inactivation in meat products and the rate of damage to the peptides from the amino acid sequence clearly indicate that bacteria and peptides are similar, in that they only require one act of exposure to irradiation to become damaged, which means that these bio-targets have uniform radiosensitivity. In contrast, different cells of pure cultures of phytopathogen *R. solani*, which can be found on the crop seed material, have variable radiobiological sensitivity, proved by the sigmoidal dose dependencies of phytopathogen inactivation. It can be noted that the efficiency of the damage to targeted pathogens and non-targeted peptides depends not only on their radiobiological properties but also on the parameters of irradiation. Thus, accelerated electrons and X-ray irradiation having different energy spectra and interaction patterns between irradiation and pathogens and surrounding molecules as well as different depth doses and LET distributions in irradiated biological objects show different efficiency of damage to both targeted microorganisms and non-targeted peptides in the doses ranges specific to each of these two groups.

### 3.3. Optimization Function to Increase Food Irradiation Efficiency

The efficiency of food irradiation consists of the maximum damage of targeted pathogens with the least damage to the surrounding non-targeted molecules, such as proteins, lipids or enzymes. This study suggests the following optimization function:

H(*D*) = ε^TM^·(1 − ε^NT^) (15)

with the maximum H_max_ allowing one to determine the optimal dose D_opt_, which can maximize the damage to the targeted microorganisms while minimizing the damage to non-targeted surrounding molecules. Since it is practically impossible to ensure that the entire volume of the irradiated food product receives the same dose, the irradiation of food products occurs within a dose range (D_min_, D_max_). In the absence of the criteria for determining the optimal dose range for industrial food irradiation, we took the approach commonly used in radiation therapy, which allows one to minimize the negative impact of irradiation to the surrounding tissues. This study suggests that the optimal dose range (D_min_, D_max_) should correspond to the values at which the optimization function H(D) runs from 0.9·H_max_ to H_max_, assuming that a value above 0.9·H_max_ would make the irradiation of food products not realistic since a narrower dose range is hardly achievable in industrial conditions. On the other hand, a value below 0.9·H_max_ would expand the dose range, causing overexposure of the food product, which would inevitably inflict harm on the surrounding non-targeted structures, deteriorating the quality of the irradiated product.

#### 3.3.1. Optimal Dose Range for Beef Irradiation

**Figure 17:**
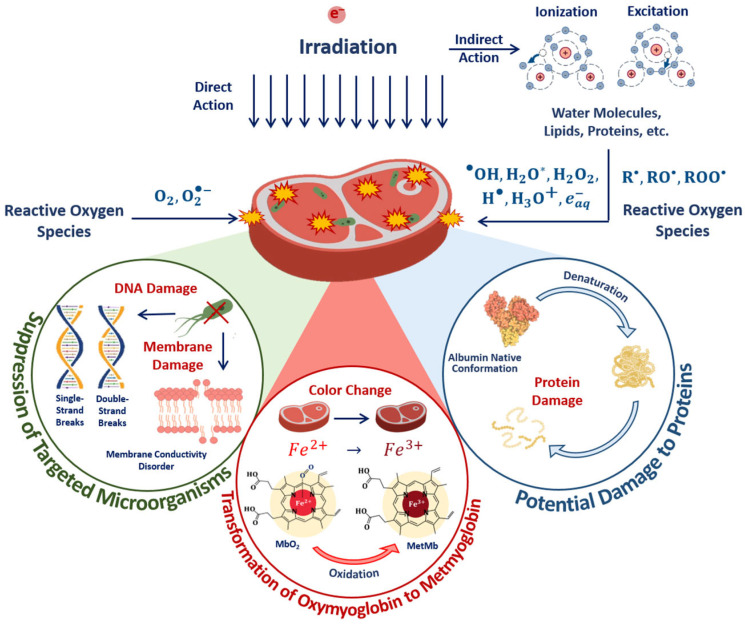
The mechanisms behind the suppression of targeted bacteria and the damage to proteins in irradiated beef.

The optimal dose range, which maximizes microorganism suppression while minimizing the impact on the surrounding molecules, such as proteins, lipids and enzymes, involves the quantitative assessment of the damage to targeted microorganisms and non-targeted surrounding molecules in the food products irradiated with different doses. The mechanisms behind the suppression of targeted bacteria and damage to surrounding molecules are determined by the irradiation dose absorbed by the beef pieces. The impact of the 1 MeV E-beam on the biological tissue of beef as a result of both the direct ionization of atoms and molecules as well as indirect action through reactive oxygen species leads to radiobiological dose effects in microorganisms and proteins (Figure 17).

In the case of direct ionization, accelerated electrons cause the destruction of bacteria cell components, such as lipids, carbohydrates and DNA, while the indirect action of accelerated electrons—another reason for bacteria suppression—leads to the formation of free radicals and reactive species, including hydroxyl radicals, hydrogen atoms and hydrated electrons, occurring as a result of water radiolysis, which interact with bacteria cell components [55]. Along with the bacteria suppression, accelerated electrons break amide bonds in proteins, leading to changes in protein structure, charge distribution and polarity of proteins. Accelerated electrons and reactive oxygen species interact with amino acids, peptides and proteins, leading to both reversible and irreversible oxidation of proteins with the formation of protein peroxyl radicals, causing disruption, modification, carbonylation, oxidation and fragmentation of the primary, secondary and tertiary structure of protein molecules [30]. As myoglobin is highly sensitive to any physical or chemical impact, transformations between myoglobin derivatives can serve as an indicator of oxidation due to the direct ionization of Fe^2+^ ions in oxymyoglobin molecules to Fe^3+^ ions and indirect action of reactive oxygen species occurring as a result of water radiolysis [56].

Considering that irradiation causes irreversible changes to the biophysical and functional properties of proteins in foods, the optimal dose range for irradiation can be determined by judging the suppression efficiency of microorganisms contaminating food products and based on the quantitative assessment of the efficiency of the damage to non-targeted proteins. This study compares the optimal dose ranges obtained while monitoring the transformations of myoglobin derivatives in beef after irradiation and through the quantitative assessment of potential damage to the albumin native structure in the suspension by determining the concentration of the selected peptides from the BSA amino acid sequence.

**Figure 18:**
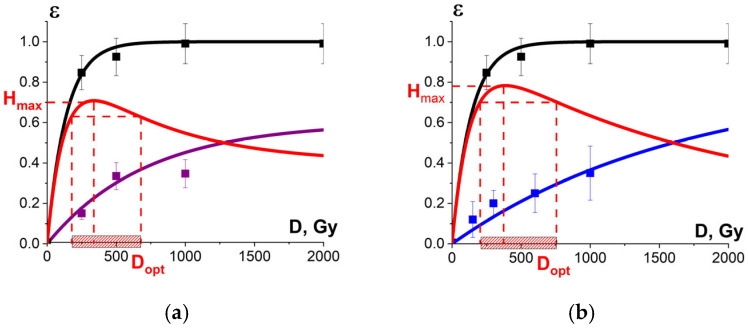
(**a**) The squares on black line show microorganism damage efficiency ε^TM^; the squares on blue line show the metmyoglobin level ε^NT^ in the beef samples irradiated with different doses. The dependencies ε^TM^(D) and ε^NT^(D) are calculated by Formula (10) with the approximation coefficients: K_1_ = 0.99 ± 0.01, *α* = (0.0073 ± 0.0003) Gy^−1^, *R*^2^ = 0.99 for microorganisms and K_1_ = 0.6 ± 0.1, *α* = (0.0014 ± 0.0003) Gy^−1^, *R*^2^ = 0.97 for metmyoglobin level, red line represents the optimization function H(D); (**b**) The squares on black line show microorganism damage efficiency ε^TM^ in the beef samples; the squares on blue line show the relative concentration of the selected peptide ε^NT^ from BSA amino acid sequence irradiated with different doses. The dependencies ε^TM^(D) and ε^NT^(D) are calculated by Formula (10) with the approximation coefficients: K_1_ = 0.99 ± 0.01, *α* = (0.0073 ± 0.0003) Gy^−1^, *R*^2^ = 0.99 for microorganisms and K_1_ = 0.9 ± 0.01, *α* = (0.00060 ± 0.00006) Gy^−1^, *R*^2^ = 0.96 for peptides. Red line represents the optimization function H(D).

Figure 18a shows the experimental dose dependencies of the microorganism suppression efficiency ε^TM^(D) and the experimental dependency of the myoglobin oxidation level *ε*^NT^(D), determined by the relative metmyoglobin concentration, on the irradiation dose measured in beef tenderloin treated with 1 MeV E-beam. Metmyoglobin concentration data, measured in the beef samples irradiated with E-beam and averaged over eight consecutive calculation repetitions, are presented in Appendix A in Table A4. As can be seen from Figure 18a, the metmyoglobin level increases with an increase in the irradiation dose absorbed by the beef samples.

Since there is a clear exponential dose dependency of the metmyoglobin level, the metmyoglobin content can be used as an irradiation marker for foods containing myoglobin, such as beef and liver. The dose dependency of the metmyoglobin level can be expressed using Formula (10). Similar to the efficiency of a microorganism suppression in beef (Figure 11) and the extent of the damage to the albumin native structure (Figure 15), the myoglobin oxidation can be manifested by factors K_1_ and K_2_. It should also be noted that myoglobin molecules did not show any signs of heterogeneity of radiobiological sensitivity K_3_.

To compare, Figure 18b shows the dose dependencies of microorganism suppression efficiency ε^TM^(D) in tenderloin beef and the dependency of the relative concentration of the selected peptide an with *m*/*z* value of 461 from the amino acid sequence of bovine serum albumin in the 0.9% saline solution on the irradiation dose ε^NT^(D), exposed to 1 MeV electron beam. Figure 18a,b show the optimization function H(D) = ε^TM^·(1 − ε^NT^) that factors in the amount of viable microorganisms and the degree of functional and structural damage to proteins: Figure 18a shows the metmyoglobin level as a marker of oxidation due to irradiation; Figure 18b shows the relative concentration of the peptide as a quantitative marker of potential damage to the native structure of proteins.

The optimal dose range obtained from myoglobin oxidation is about 220–854 Gy, which is quite similar to the optimal dose range (204–755 Gy) obtained from the structural damage of albumin. Since this study explores myoglobin oxidation and the potential damage to the albumin native structure as a side effect of irradiation on the non-targeted surrounding proteins, the overlapping of the two optimal dose ranges ensures that no negative impact of irradiation on beef proteins having a high nutritional value is detected following irradiation with 1 MeV accelerated electrons.

#### 3.3.2. Optimal Dose Range for Seed Potato Irradiation

To find the optimal dose range for pre-planting electron beam irradiation of seed potato tubers with natural infection by fungal diseases, we studied the impact of irradiation on targeted phytopathogens *R. solani*, which can be found on the surface of the tubers and non-targeted potato sprouts. We monitored the germination rate and the total potato yield to establish the dose range at which the negative impact on the growth and development of plants is minimized while improving the quality of the yield as well as the phytosanitary condition of agrocenosis.

**Figure 19:**
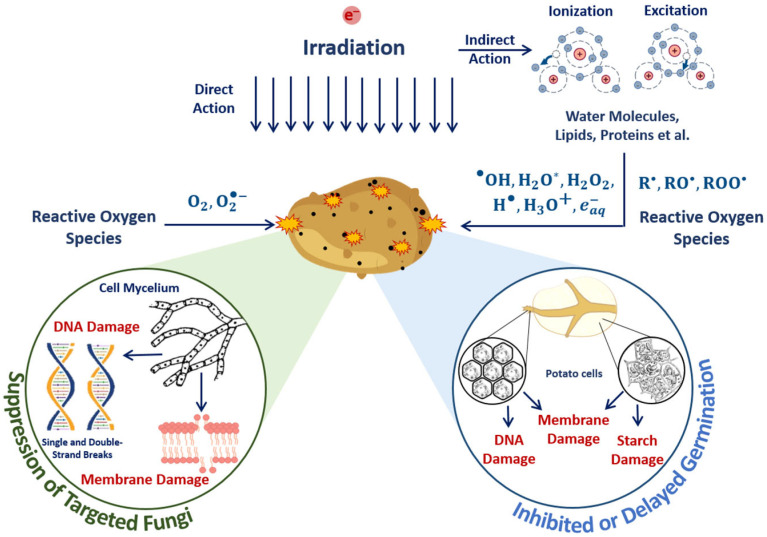
Mechanisms of direct ionization of seed potato components and indirect action on plant cells through reactive oxygen species during E-beam irradiation.

The suppression of targeted phytopathogens and damage to potato structures in surface layers containing sprouts, flesh cells and starch damage to surrounding molecules are determined by the mechanisms of direct ionization of seed potato components and indirect action on plant cells through reactive oxygen species (Figure 19). Irradiation inhibits tuber germination, and the higher the dose, the more inactivated cells appear after irradiation.

**Figure 20:**
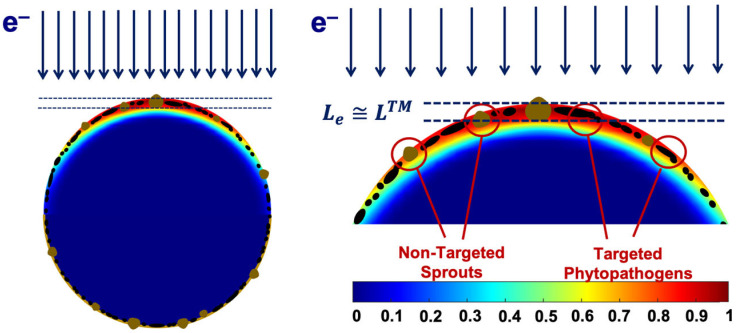
Depth dose distribution map with data calculated using GEANT 4 toolkit for a 40 mm spherical water phantom irradiated with 1 MeV electron beam.

We found that the 1 MeV electron beam is suitable for the treatment of seed potato tubers naturally infected with fungal diseases since the penetration depth *L*_e_ of 1 MeV electrons of up to 5.5 mm is sufficient for inhibiting phytopathogens that can be found at a depth of up to *L*^TM^ = 2–5 mm. The extent of the phytopathogen suppression within the surface layers of potato tubers is largely determined by the number of ionization events hitting fungi cell components. Figure 20 below shows the color distribution of the relative dose absorbed by a 40 mm spherical water phantom, which simulates a potato tuber irradiated with 1 MeV electrons. As can be seen, the maximum exposure shown in red occurs at a depth of 2 mm, where the overwhelming majority of targeted phytopathogens can be found. Considering that the IAEA recommends using low-energy electron beams and low-energy bremsstrahlung radiation [1,2], the 1 MeV E-beam can be viewed as highly efficient and reliable for agricultural use.

**Figure 21:**
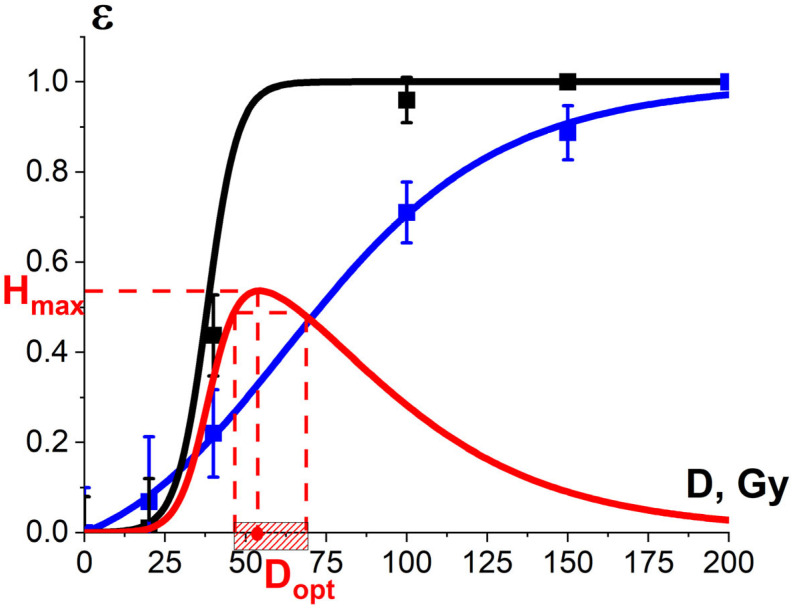
The squares on the black line show the dose dependency of the damage efficiency $\varepsilon_{exp}^{TM}\left(D\right)$ for phytopathogen *R. solani* calculated using Formula (13); the squares on the blue line show the dose dependency of the potato yield losses $\varepsilon_{exp}^{TM}\left(D\right)$; ε^TM^(D) and *ε*^NT^(D) dose dependencies are calculated using Formula (11). These data are provided for irradiation with accelerated electrons. Approximation of experimental data by Formula (11) was performed with the approximation coefficients: $K_{1}=1,\;\bar{D}=(38.0\pm 0.3)$ Gy, σ = (4.6 ± 0.5) Gy, R^2^ = 0.99 for *R. solani*; $K_{1}=1,\;\bar{D}=(53.0\pm 0.8)$ Gy, σ = (11.0 ± 0.7) Gy, R^2^ = 0.99 for the potato yield. Red line represents the optimization function H(D).

Figure 21 shows the experimental dependency of the *R. solani* suppression efficiency $\varepsilon_{exp}^{TM}\left(D\right)$ in potatoes, which is estimated as the decrease in the diameter of fungi taken from the surface of irradiated seed potato tubers in relation to that taken from the surface of non-irradiated tubers, on the irradiation dose. Since the dependency $\varepsilon_{exp}^{TM}\left(D\right)$ is sigmoidal, it can be approximated using Formula (13). As can be seen from Figure 13 and Figure 21, while 1 MeV E-beam irradiation of pure cultures of phytopathogens is only able to fully suppress *R. solani* at doses of 5000 Gy and higher, the 1 MeV electron beam at doses of 100 Gy and higher eliminates *R. solani* in the surface layers of seed potato tubers. It should be noted that the dependency of the *R. solani* suppression efficiency $\varepsilon_{exp}^{TM}\left(D\right)$ in potatoes on the irradiation dose is sigmoidal and can be described by Formula (11).

Since it was found that the potato yield decreases with an increase in the dose absorbed by the seed potato tubers, which is a sign that irradiation has a negative impact not only on the phytopathogens but also on the potato sprouts on the potato surface, we monitored and estimated the losses in the yield using the following formula:

(16) $$\varepsilon_{exp}^{NT}=1-\frac{\bar{Y_{irr}}}{\bar{Y_{ref}}},$$

where $\bar{Y_{irr}}$ and $\bar{Y_{ref}}$ are the average amounts of potato yield grown from irradiated and non-irradiated seed potatoes, respectively, to determine the optimal dose range for seed potato irradiation. The data on the potato yield grown from irradiated and non-irradiated tubers are presented in Appendix A in Table A5. As Formula (16) suggests, the dependency of the potato yield $\varepsilon_{exp}^{NT}(D)$ on the irradiation dose absorbed by the seed potatoes is sigmoidal (Figure 21) and can be described by Formula (11). Therefore, the potato sprouts have heterogeneous radiobiological sensitivity, which reveals the influence of K_3_ factor on the potato sprout suppression.

As can be seen from Figure 21, the optimal dose range at which the optimization function H(D) runs from 0.9·H_max_ to H_max_ is 46–67 Gy. Considering that potato varieties have different potato sprout depths and biochemical properties, such as water and starch content, they call for a different optimal dose range to suppress phytopathogens to the required level while minimizing the negative impact on the potato yield.

It should be noted that the limits of the optimal dose range depend not only on the irradiation parameters, such as the type of irradiation, energy spectrum and dose uniformity, but also on the physical and chemical properties of the food product, such as water and oxygen content, ambient temperature around the irradiated food product as well as storage temperature. Moreover, the optimal dose range can shift depending on the focus on the particular changes occurring in the surrounding molecules as a result of irradiation, which depend on the irradiation dose. Also, the limits of the optimal dose can change due to the variety of microorganisms found in the food products as well as the method used to detect microorganisms present in irradiated food as a result of natural contamination.

## 4. Conclusions

The comprehensive food irradiation optimization method involving the optimization function H(D) allows one to establish the optimal dose range for each category of plant and animal product that suppresses targeted pathogens while preserving molecules and cells that determine the nutritional value of the products. The function H(D) takes into account irradiation dose uniformity throughout the irradiated food product, the probability of irradiation hitting microorganisms and surrounding molecules in the food product as well as the heterogeneity of the radiobiological sensitivity of pathogens and biological structures.

In the absence of specific criteria setting out the lower and upper limits of the irradiation dose range for food products with a complex composition, the geometry as well as specific pathogens distributed differently depending on the type of product, we conducted this research to study how a plant product and an animal product respond to irradiation using seed potato tubers and tenderloin beef as examples. It was confirmed that the optimal dose range for animal product is significantly higher than that for plant product since the nature of bacteria and their distribution throughout the whole volume of the product require a higher probability of electrons hitting the critical structures of every pathogen cell. Plant products in which targeted pathogens can be primarily found on the surface, however, call for a highly precise dose range determined by the specific properties of a particular plant product, which ensures the exposure of the surface layers to the maximum required irradiation dose while minimizing the impact of irradiation on the internal layers of the product containing critical biological structures responsible for germination, yield and the quality of plant products.

Considering that food irradiation has different objectives and goals, it is necessary to determine the optimization function for each specific case, taking into account the individual properties of the food product before estimating the optimal irradiation parameters, which would ensure targeted irradiation of the product, without causing a detrimental effect on its organoleptic properties. The precision of the proposed optimization function can be further enhanced by exploring the potential of a wider range of irradiation markers beyond the metmyoglobin level and the concentration of the selected peptides from the amino acid sequence of albumin, which we established for the use in the optimization function.

## Figures and Tables

**Table 1:** Tuber irradiation specification.

Session Number	Total Time of Irradiation from Two Sides, s	Beam Current, µA	Charge on the Plate *Q_exp_*, nC	Absorbed Dose *D*, Gy
1	32 ± 1	0.10 ± 0.01	2070 ± 40/2070 ± 40	20.0 ± 0.4
2	50 ± 1	0.10 ± 0.01	4120 ± 70/4080 ± 70	40 ± 1
3	100 ± 1	0.10 ± 0.01	10,170 ± 200/10,240 ± 200	100 ± 2
4	150 ± 1	0.10 ± 0.01	15,430 ± 300/15,220 ± 300	150 ± 3
5	200 ± 1	0.10 ± 0.01	20,320 ± 400/20,360 ± 400	200 ± 4

## Data Availability

The original contributions presented in this study are included in the article; further inquiries can be directed to the corresponding author/s.

## Appendix A

**Table A1:** The number of viable cells irradiated with electron beam and X-ray irradiation and non-irradiated beef homogenate.

Number of Viable Cells, CFU/g and Averaged over Six Consecutive Calculation Repetitions		
Dose, Gy	E-Beam	X-Ray
0	(1.0 ± 0.2) × 10^4^	(1.0 ± 0.2) × 10^4^
250	(0.15 ± 0.03) × 10^4^	(0.5 ± 0.13) × 10^4^
500	(0.05 ± 0.014) × 10^4^	(0.21 ± 0.03) × 10^4^
1000	(0.018 ± 0.003) × 10^4^	(0.020 ± 0.007) × 10^4^
2000	(0.014 ± 0.002) × 10^4^	(0.021 ± 0.002) × 10^4^
3000	(0.019 ± 0.003) × 10^4^	(0.009 ± 0.005) × 10^4^

**Table A2:** The diameters of fungi grown from irradiated and non-irradiated mycelium cells averaged over three consecutive calculation repetitions.

Dose, Gy	Diameter of Fungi Colonies, mm
0	40.1 ± 3.2
100	40.3 ± 4.4
1000	16.9 ± 3.6
5000	0
10,000	0

**Table A3:** Data on the concentration of the peptide having *m*/*z* value of 461 averaged over five consecutive calculation repetitions in BSA suspensions irradiated with E-beam and X-rays at doses ranging from 150 Gy to 8000 Gy.

Concentration of Peptide, %			
Dose, Gy	X-Ray	Dose, Gy	E-Beam
0	99.0 ± 0.9	0	100 ± 1.0
150	87.3 ± 1.6	150	88.0 ± 0.9
300	78.1 ± 1.2	300	80.0 ± 1.5
450	83.5 ± 3.1	600	75.0 ± 1.8
600	58.4 ± 3.3	1000	65.0 ± 2.6
1000	55.9 ± 6.2	4000	31.0 ± 5.0
4000	17.3 ± 7.2	8000	16.0 ± 6.0
6000	3.0 ± 7.4	-	-
8000	1.1 ± 0.1	-	-

**Table A4:** Metmyoglobin concentration data measured in the beef samples irradiated with E-beam and averaged over eight consecutive calculation repetitions.

Dose, Gy	Metmyoglobin Concentration, %
0	100
250	84.9 ± 3.0
500	66.5 ± 6.7
1000	65.3 ± 6.9
5000	55.8 ± 8.8
10,000	50.0 ± 10.0

**Table A5:** The data on the potato yield grown from irradiated and non-irradiated tubers.

Dose, Gy	Potato Yield, tons/hectare
0	26.5 ± 2.7
20	24.7 ± 3.8
40	20.7 ± 2.6
100	7.7 ± 1.8
150	3.0 ± 1.6
200	0
